# Pigmented paravenous retinochoroidal atrophy associated with Vogt-Koyanagi-Harada disease: a case report

**DOI:** 10.1186/s12886-020-1318-4

**Published:** 2020-01-29

**Authors:** Prithvi Ramtohul, Alban Comet, Pierre Gascon, Danièle Denis

**Affiliations:** Centre Hospitalier Universitaire de l’Hôpital Nord, chemin des Bourrely, 13015 Marseille, France

**Keywords:** Pigmented paravenous retinochoroidal atrophy, Retinitis pigmentosa, Spectral domain optical coherence tomography, Vogt-Koyanagi-Harada

## Abstract

**Background:**

To describe a unique case of pigmented paravenous retinochoroidal atrophy that developed several years after Vogt-Koyanagi-Harada disease.

**Case presentation:**

A 28-year-old woman presented with gradual vision loss in both eyes and nyctalopia for 2 years. Past medical history was relevant for Vogt-Koyanagi-Harada disease since the age of 19 and positive HLA-DR4. Funduscopic examination revealed perivascular pigmentary clumping and atrophic changes radiating from the optic disks. Spectral domain optical coherence tomography through the macula demonstrated perifoveal outer retinal layers loss with cystic degeneration. Fundus autofluorescence showed zonal areas of hypoautofluorescence corresponding to the areas of atrophy. Full-field electroretinogram identified mildly reduced scotopic and photopic responses. The patient was diagnosed with pigmented paravenous retinochoroidal atrophy.

**Conclusions:**

Pigmented paravenous retinochoroidal atrophy may be acquired after Vogt-Koyanagi-Harada disease. Pathogenesis of pigmented paravenous retinochoroidal atrophy may involve inflammatory-related precursors on a background of genetic predisposition.

## Background

First termed retinochoroiditis radiata in 1937, pigmented paravenous retinochoroidal atrophy (PPRCA) is a rare form of chorioretinal atrophy characterized by perivenous retinal pigment epithelial atrophy and pigment clumping [1]. It is typically bilateral, symmetric and non-progressive or slowly progressive and is most often discovered incidentally during routine fundus examination. Vision is generally normal or mildly reduced at presentation, with minimal to no progression over time [2]. Although the etiology of PPRCA remains unknown, there are several hypotheses including genetic, inflammatory or infectious causes. Heterozygous *CRB1* variant of uncertain significance in a family with dominantly inherited PPRCA and variable expressivity has been reported [3]. Similarly, tuberculosis, congenital syphilis, Behçet disease, measles and rubella have been proposed as causative [4].

We reported a unique case of PPRCA occurring in a background of Vogt-Koyanagi-Harada disease and raised pathophysiologic hypothesis based on optical coherence tomography findings.

## Case presentation

**Fig. 1:**
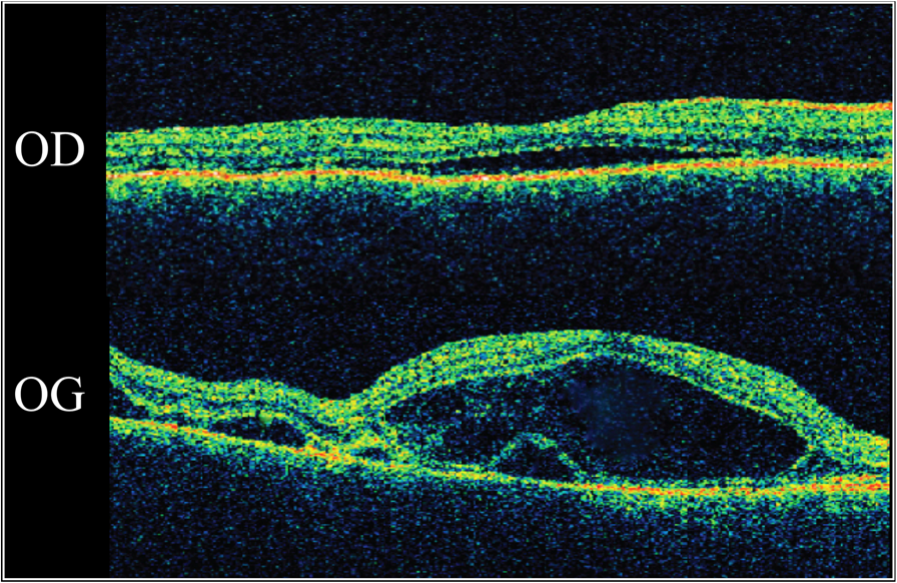
Initial time domain optical coherence tomography at the age of 19 showed serous retinal detachments with subretinal septa consistent with acute Vogt-Koyanagi-Harada disease

A 28-year-old woman was referred for gradual vision loss in both eyes and nyctalopia for 2 years. Past medical history was relevant for Vogt-Koyanagi-Harada (VKH) disease since the age of 19 and positive HLA-DR4 (Fig. 1). She was treated with systemic corticosteroids administered orally at a dose of 1 mg/kg/day and azathioprine (1 mg/kg/day) for 1 year. No recurrences were observed and the patient was lost to follow-up.

**Fig. 2:**
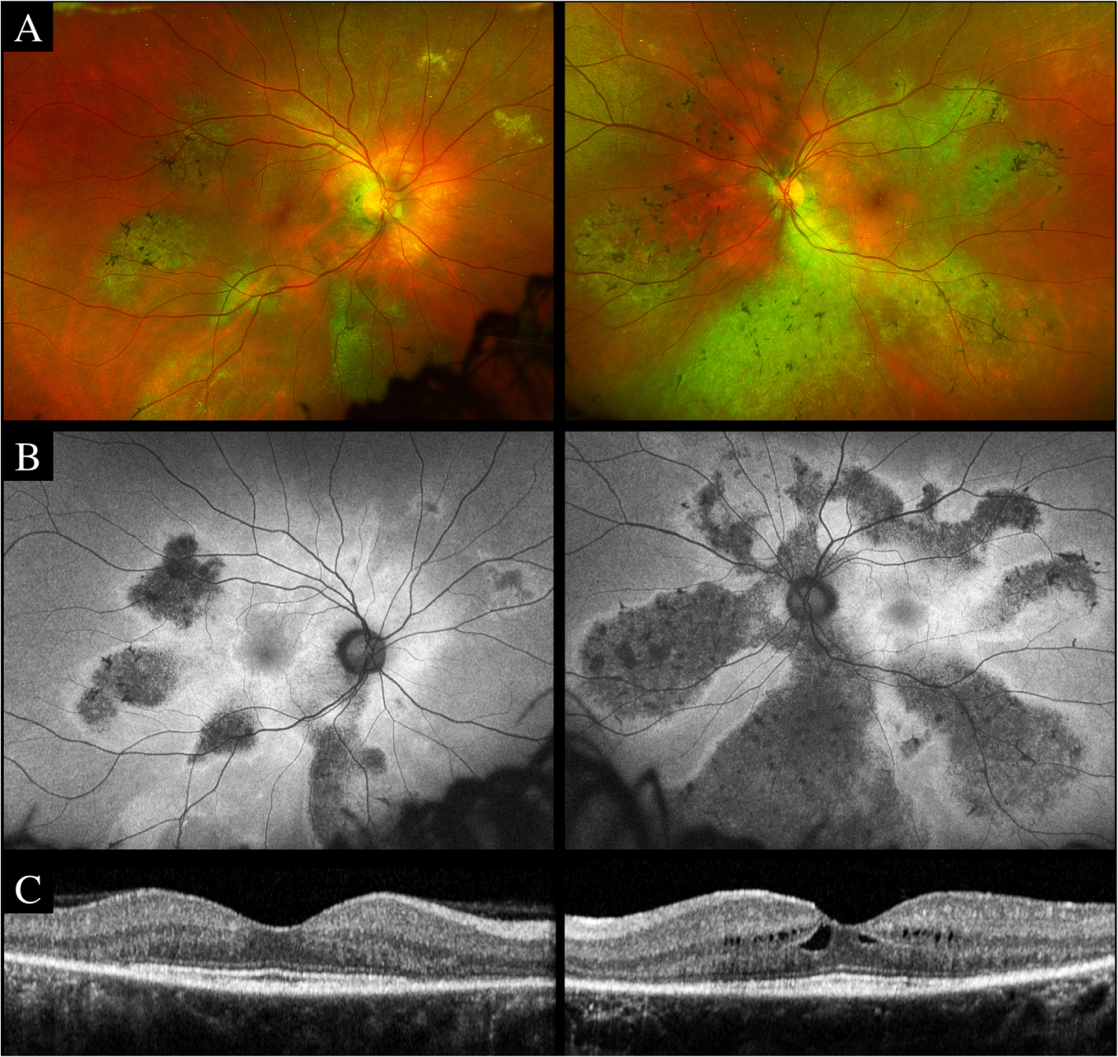
**a**. Ultra-widefield fundus photography (Optos, Dunfermline, UK) demonstrated zonal areas of retinochoroidal atrophy distributed along the retinal veins with bone-spicule pigmentation and sparing of the macula. **b**. Fundus autofluorescence (AF) (Optos, Dunfermline, UK) showed zonal areas of hypoAF along the retinal veins surrounded by linear hyperAF edges. **c**. Spectral domain optical coherence tomography (Spectralis, Heidelberg Engineering, Heidelberg, Germany) through the macula demonstrated perifoveal outer nuclear layers thinning, ellipsoid and interdigitation zones disruption and cystic degeneration in both eyes

**Fig. 3:**
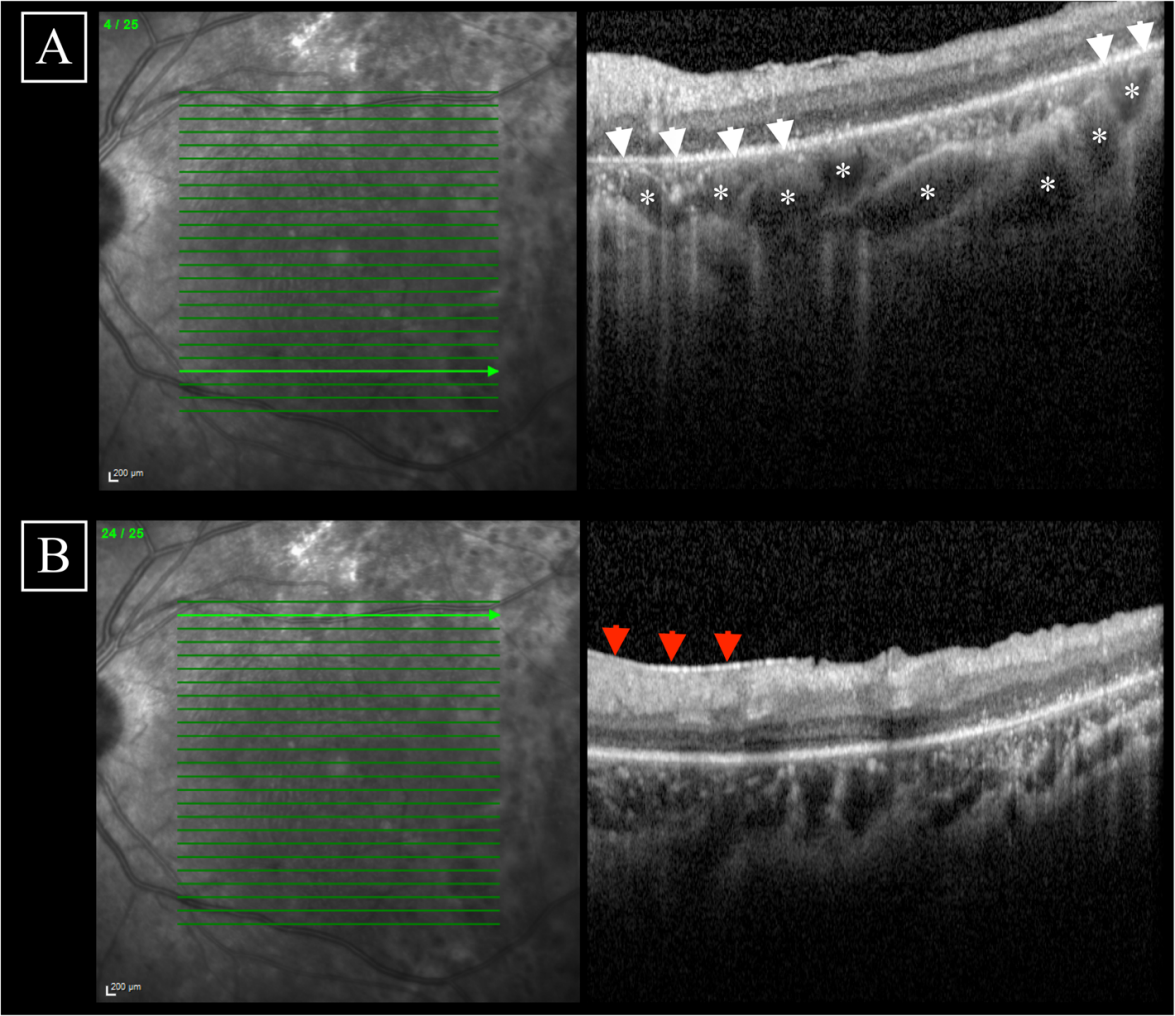
**a**. Spectral domain optical coherence tomography (SD-OCT) showed extensive choroidal changes with choriocapillaris and inner choroidal atrophy (red arrowheads) with persistent Haller layer’s vessels (asterisks). Note the mark penetration of optical coherence tomography infrared light through the areas of retinal pigment epithelium loss. **b**. SD-OCT demonstrated thickening and cystic degeneration of the retinal nerve fiber layer (white arrowheads)

At current presentation, best-corrected visual acuity was 20/25 in both eyes. Anterior segment examination was unremarkable. Funduscopic examination revealed circumscribed areas of retinochoroidal atrophy and pigment clumping along the retinal veins (Fig. 2a). Fundus autofluorescence (AF) showed zonal areas of hypoAF along the retinal veins surrounded by linear hyperAF edges (Fig. 2b). Spectral domain optical coherence tomography (SD-OCT, Spectralis, Heidelberg Engineering, Heidelberg, Germany) through the macula demonstrated perifoveal outer retinal layers disruption with cystic degeneration in both eyes (Fig. 2c). SD-OCT through the atrophic lesions highlighted loss of the outer retinal layers, retinal pigment epithelium (RPE) atrophy, and extensive inner choroidal thinning with preservation of the Haller’s layer vessels (Fig. 3a). Perivenular SD-OCT scan revealed thickening of the retinal nerve fiber layer (RNFL) (Fig. 3b). Full-field electroretinogram identified mildly reduced scotopic response and a slight decrease in b-wave amplitude (photopic response). The patient was diagnosed with pigmented paravenous retinochoroidal atrophy.

## Discussion and conclusions

PPRCA is a rare disease characterized by perivenous aggregations of pigment clumping associated with zonal areas of retinochoroidal atrophy distributed along the retinal veins. Patients are typically asymptomatic and the disease process is non-progressive or slowly progressive [1]. The underlying etiology of PPRCA remains controversial and a hereditary nature appeared to be a reasonable assumption since McKay et al. reported a dominantly inherited PPRCA in a family with a heterozygous *CRB1* variant [3]. Similarly, Obata et al. described PPRCA in two Japanese siblings with variable expressivity [5]. However, in a recent large-cohort study of 23 patients, Shona et al. found no evidence of a genetic basis because 96% of patients had no familial history of inherited ocular disease [6]. Interestingly, there a several evidence in the literature reporting PPRCA onset after an episode of intraocular inflammation, including tuberculosis, congenital syphilis, Behçet disease, measles and rubella immunization or vaccination [6]. To our knowledge, we report the first case of PPRCA associated with VKH disease and positive HLA-DR4. This further raises the possibility of triggering inflammatory events on a background of genetic predisposition.

Multimodal imaging techniques have led to better characterization of PPRCA [7]. In our case, geographic areas of RPE loss on SD-OCT corresponded to areas of reduced AF and thinning of the outer retinal layers colocalized with the linear increased AF. We also documented cystic macular edema (CME) which has exceptionally been described in PPRCA [6]. The authors postulated that the underlying pathogenesis may be comparable to retinitis pigmentosa-associated CME, including blood-retinal barrier breakdown, dysfunction of the RPE pumping function, Müller cells failure and vitreous traction [8]. Intriguingly, SD-OCT scan through the atrophic lesions showed perivenular thickening of the RNFL. This observation has been reported only in one case [9]. This singular SD-OCT feature is of physiopathological interest; in fact, it was hypothesized that choroidal atrophy resulting in decreased choroidal perfusion and subsequent insufficient metabolic supply for the outer retinal layers preceded the photoreceptors degeneration and then the RPE loss [10]. Perivascular thickening of the RNFL may support an additional vessel-based etiology for PPRCA rather than a RPE-based mechanism only. It has to be noted that chronic stages of VKH disease may present with several patterns of chorioretinal atrophy, including peripapillary atrophy, multiple nummular atrophic scars, irregular areas of atrophy and sectoral chorioretinal atrophy, which could have masqueraded as PPRCA [11]. In our case, the localized areas of retinal disturbance with normal chorioretinal paravenous zone in between lesions further suggest PPRCA diagnosis. Conversely, Huang et al. suggested that PPRCA following inflammatory retinal vein vasculopathy, resulting from various causes previously cited, are not real PPRCA and should be termed pseudo PPRCA [12]. In fact, a previous medical history is rarely found and appears to be a confounding rather than a causative factor [13].

## Conclusion

In conclusion, we reported the first case of PPRCA occurring in a background of Vogt-Koyanagi-Harada disease. We highlighted multimodal imaging correspondence of this rare pathology and raised pathophysiologic hypothesis based on optical coherence tomography features.

## Data Availability

All data generated or analysed during the current study are included in this published article.

## Abbreviations

AF: Fundus autofluorescence
CME: Cystic macular edema
PPRCA: Pigmented paravenous retinochoroidal atrophy
RNFL: Retinal nerve fiber layer
RPE: Retinal pigment epithelium
SD-OCT: Spectral domain optical coherence tomography
VKH: Vogt-Koyanagi-Harada
